# Recent advances in the epidemiology, clinical and diagnostic features, and control of canine cardio-pulmonary angiostrongylosis

**DOI:** 10.1186/s13567-014-0092-9

**Published:** 2014-09-27

**Authors:** Hany M Elsheikha, Sarah A Holmes, Ian Wright, Eric R Morgan, David W Lacher

**Affiliations:** School of Veterinary Medicine and Science, University of Nottingham, Sutton Bonington Campus, Leicestershire, LE12 5RD UK; Withy Grove Veterinary Surgery, 39 Station Rd, Bamber Bridge, Preston, PR5 6QR UK; School of Veterinary Science, Langford House, University of Bristol, Langford, North Somerset BS40 5DU UK; Division of Molecular Biology, Center for Food Safety and Applied Nutrition, United States Food and Drug Administration, Laurel, MD USA

## Abstract

The aim of this review is to provide a comprehensive update on the biology, epidemiology, clinical features, diagnosis, treatment, and prevention of canine cardio-pulmonary angiostrongylosis. This cardiopulmonary disease is caused by infection by the metastrongyloid nematode *Angiostrongylus vasorum*. The parasite has an indirect life cycle that involves at least two different hosts, gastropod molluscs (intermediate host) and canids (definitive host). *A. vasorum* represents a common and serious problem for dogs in areas of endemicity, and because of the expansion of its geographical boundaries to many areas where it was absent or uncommon; its global burden is escalating. *A. vasorum* infection in dogs can result in serious disorders with potentially fatal consequences. Diagnosis in the live patient depends on faecal analysis, PCR or blood testing for parasite antigens or anti-parasite antibodies. Identification of parasites in fluids and tissues is rarely possible except post mortem, while diagnostic imaging and clinical examinations do not lead to a definitive diagnosis. Treatment normally requires the administration of anthelmintic drugs, and sometimes supportive therapy for complications resulting from infection.

## Table of contents

1. Introduction
2. Historical perspective
3. Biology
  3.1. Life-cycle of *A. vasorum*
  3.2. Morphological characteristics
  3.3. Host-specificity
4. Epidemiology
  4.1. Pattern of spread
  4.2. Risk factors and at risk-populations
  4.3. Role of wildlife reservoirs
5. Disease clinical features
  5.1. Pulmonary
  5.2. Coagulopathy
  5.3. Cardiovascular
  5.4. Nervous
6. Diagnosis of angiostrongylosis
  6.1. Laboratory diagnosis
    6.1.1. Pathological findings
    6.1.2. General laboratory findings
    6.1.3. Parasitological findings
  6.2. Immuno-diagnosis
    6.2.1. Antigen-based assays
    6.2.2. Antibody-based assays
  6.3. Molecular techniques
  6.4. Problems in diagnosing angiostrongylosis
7. Treatment
8. Control and prevention
9. Conclusions
10. Competing interests
11. Authors’ contributions
12. Acknowledgements
13. References

## 1. Introduction

As a disease with a substantial animal health impact, canine angiostrongylosis remains a high priority for clinicians and researchers. Infected dogs usually exhibit signs of respiratory and/or cardiovascular disease, and occasionally coagulopathies and neurological signs, with fatal consequences in severe cases [1,2]. This disease is caused by the metastrongyloid nematode *Angiostrongylus vasorum* (Baillet, 1866) Kamensky, 1905 (Nematoda: Metastrongylidae). Even though *A. vasorum* was discovered over a century ago in France [3] knowledge of its epidemiology and clinical importance has grown in the last 20 years. In this time, infection has dramatically increased in significance and apparent incidence, and the parasite has expanded from established “hotspots” into previously uninfected areas. Popular theories include climatic change, increased urbanisation of the red fox (*Vulpes vulpes*) which acts as a reservoir host [4,5] and increased movement of domestic dogs both within and between countries [6]. However, the spread of the parasite is likely due to a combination of these and possibly other factors, and this makes infection with *A. vasorum* unpredictable.

Extremely valuable clinical observations have been obtained in previous studie*s* [7-11], including studies examining moderate numbers of animals, e.g. 160 cases [1] and 54 cases [12], which document trends in the signalment of presenting animals. However, more studies with larger sample sizes in a range of settings are needed in order to draw robust conclusions. The use of signalment trends is valuable to veterinary practitioners, as it allows improved education of dog owners and perhaps facilitates faster diagnosis. Useful information includes identification of at-risk breeds, or breed groups, as well as some knowledge regarding the degree of influence that the sex or age of an animal has upon likelihood of infection. Likewise, knowledge of the way in which *A. vasorum* affects physical parameters, haematological and biochemical parameters assists practitioners in compiling a list of differential diagnoses. When a diagnosis of *A. vasorum* has been established it is useful for practitioners to have additional information regarding the most effective treatment strategies.

Over the last few decades *A. vasorum* has not escaped the interest of veterinary clinicians and parasitologists. This is reflected in the variety and growing number of scientific publications, which continue to advance the knowledge on this parasite and the disease it causes. Hence, we here review the seminal literature and summarise the latest advances in the epidemiology, clinical, and laboratory diagnostics of *A. vasorum* infection, and discuss current treatment and prevention strategies. Additionally, the historical perspective, biology, morphological characteristics and host specificity are discussed.

## 2. Historical perspective

The name *A. vasorum* was derived from the Greek words “angion” meaning bottle, “strongylos” meaning round, and “vasorum” meaning blood vessels. The pseudonym “French Heartworm”, by which *A. vasorum* is commonly known, was given based on the first locality from which the parasite was recognized. When studying historical accounts relating to the distribution of *A. vasorum* in Toulouse, France in the 1800s to various countries throughout Europe, Africa and the Americas to date, the nematode appears to have spread at an alarming rate and to a wide geographical expanse. Thus, from its initial recognition in France, *A. vasorum* has been described in bordering countries within Northern Europe, whereby infection appeared to establish within well-defined endemic foci. There were also reports of *A. vasorum* infection within canid populations outside of Europe, more specifically in South America, Newfoundland in North America, and Uganda [1,13]. For many years, very few cases of infection were documented outside of these “hotspots”. More recent studies have begun to highlight an apparent change in the range of *A. vasorum*, reporting instances of infection from new localities a considerable distance from the previously recognised foci [1,14,15].

## 3. Biology

### 3.1. Life-cycle of *A. vasorum*

The dioecious adult *A. vasorum* lives in the pulmonary arteries and heart of the definitive hosts, i.e. dogs and other canids, and produces eggs that hatch to first-stage larvae (L1). These penetrate the alveoli, migrate up to the oropharynx, after which they are swallowed and then eliminated in the faeces. A definitive host can shed as many as 280 000 larvae per gram of faeces [16]. These L1 must infect a gastropod mollusc intermediate host (slug or snail), for the development of the infective third-stage larvae (L3), which occurs within 10-16 days under optimum conditions [1,14,17]. Until now virtually all investigated snails and slugs have proven to be able to function as intermediate host to this parasite. Frogs act as paratenic hosts, following the ingestion of infected snails or slugs, and can also act as intermediate hosts [18]. L3 of *A. vasorum* are able to infect the dog (*Canis familiaris*) either directly, through ingestion of L3 present in the environment, or indirectly, through ingestion of an intermediate gastropod or paratenic (transport) host. Then, L3 penetrate the gut wall and migrate to the abdominal lymph nodes, where they moult to fourth-stage larvae (L4); then enter the portal circulation, migrate through the liver parenchyma and eventually reach the right ventricle and pulmonary arteries, where they mature to adults. The time from which a definitive host is infected to when larvae can be recovered from faeces, otherwise known as the pre-patent period, is reported to be 38–57 days; however, it can range widely from 28 to 108 days [19].

To date, no work has been done to determine whether infection status affects longevity of molluscs experimentally infected with *A. vasorum*. The survival of *A. vasorum* larvae was found to be greater at lower temperatures, with larvae remaining active until day 15 and alive until day 24 at 5°C, while high temperatures can induce higher mortality [20]. A seasonal pattern to infection by *A. vasorum* has been detected in dogs in Denmark [10]. A study carried out by Morgan et al., [4] approximated an eco-climatic index to predict the success of *A. vasorum* establishment and growth in different climatic locations. The index correlated a high eco-climatic index with areas known to have high *A. vasorum* infection, thus highlighting a potential use to anticipate infection in currently non-endemic areas.

### 3.2. Morphological characteristics

**Figure 1:**
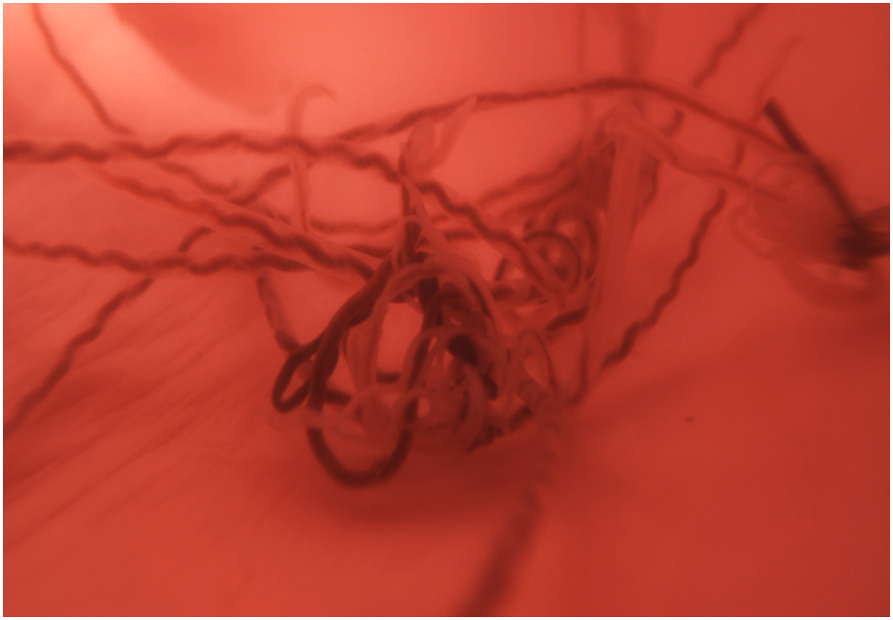
**Mimicking of adult** ***Angiostrongylus vasorum in situ*** **using reconstructive filming.** Note the characteristic barber’s pole appearance of female worms in a still image taken from a filmed sequence of adult live worms, which were collected from naturally infected dogs and placed in a pig’s heart through which fluid was pumped.

**Figure 2:**
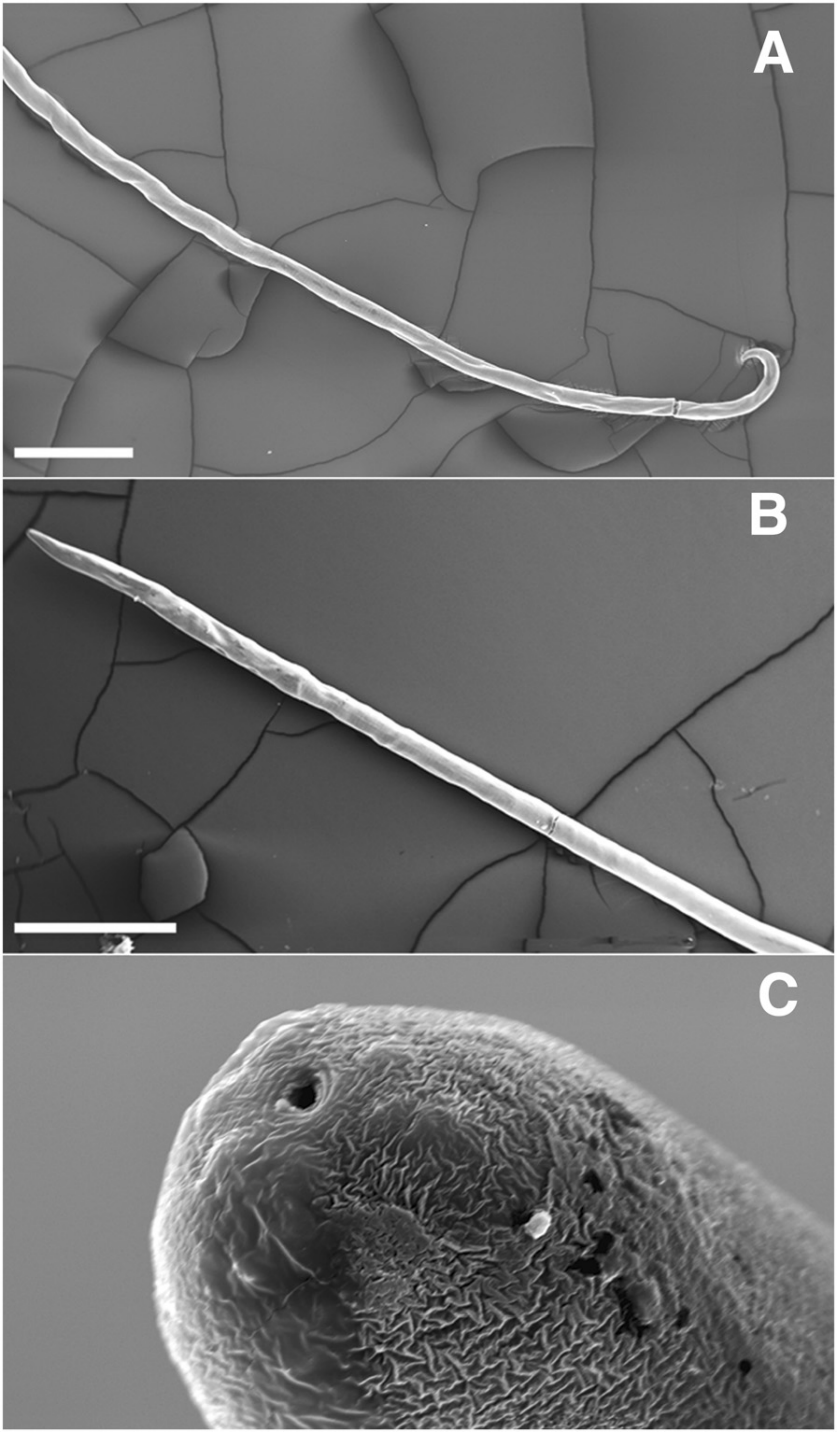
**SEM photograph of adult** ***Angiostrongylus vasorum*** **. (A)** Caudal region, **(B)** anterior region, **(C)** a higher magnification of the anterior end showing the oral orifice. Scale bars; A & B = 1 mm; C = 10 μm.

**Figure 3:**
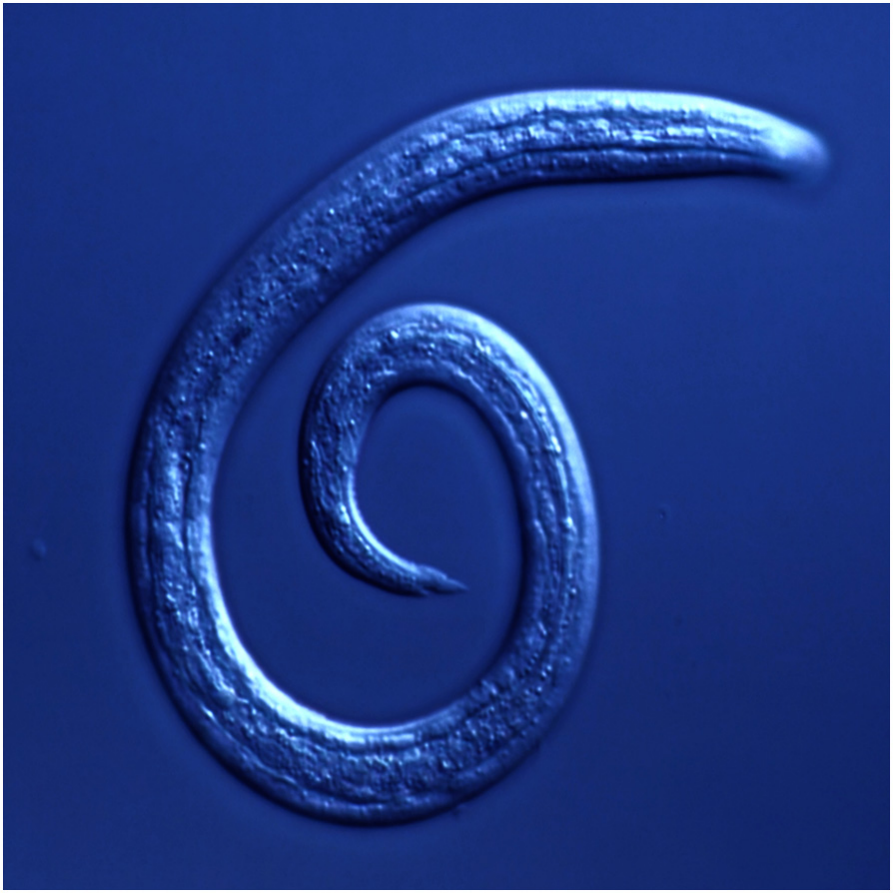
***Angiostrongylus vasorum*** **first-stage larva recovered from faeces using the Baermann technique.** Note the characteristic kinked tail of the larva with a sinusoidal curve and a spine and distinct notch on the dorsal surface.

The basic morphological appearance of *A. vasorum* is like that of most nematodes, taking a cylindrical form that tapers at each end. Members of the superfamily Metastrongyloidea, to which *A. vasorum* belongs, are all known to feature a small buccal capsule, rather than just a simple oral aperture. Distinguishing features that allow specific identification of *A. vasorum* can be split into gross and microscopic categories and characterized by sex and larval stage. Males are 14-18 mm in length, with visible bursa, spicules and bursal rays, their ventral rays are fused for the majority of their body and the dorsal ray is short with short terminal branches. Females are slightly larger, 18-25 mm in length, and have a gross appearance similar to the “barber’s pole” appearance of *Haemonchus* sp. The white ovaries of female *A. vasorum* are wrapped in a coil around the intestine with the vulva located in the posterior region of the body (Figures 1 and 2). The undifferentiated eggs that hatch from the oviparous female develop into L1 larvae (Figure 3) that are 280 to 330 μm in length, have an anterior cephalic button, a dorsal spine and a sinus wave curve in their tail [17,21]. Knowledge of L1 morphology is essential for diagnosis using Baermann’s technique.

### 3.3. Host-specificity

It is now recognised that *A. vasorum* infection is commonly seen in wild canids, such as red foxes [22], which act as reservoir hosts, and can also infect the wolf (*Canis lupus*), coyote (*Canis latrans*), and jackal (*Canis aureus*), as well as other wild canid species [23-25]. A correlation between the prevalence of *A. vasorum* infection in foxes and in dogs has been suggested. This connection has led to studies exploring other potential reservoirs of infection that may threaten the dog population. New potential definitive hosts, such as the Eurasian badger (*Meles meles*) were identified [26,27]. Also, natural *A. vasorum* infection has been reported in captive red panda (*Ailurus fulgens*) in the United Kingdom and Denmark [28,29] and in free living otters in Denmark [30].

## 4. Epidemiology

*A. vasorum* is recognised as having a worldwide distribution. But, when focussing on the enzootic prevalence of *A. vasorum* globally there are limited data from which to draw conclusions. No national or international surveillance mechanisms are in place to determine the prevalence and global distribution of *A. vasorum* infection. The geographic distribution of *A. vasorum* in the Americas and Africa is less defined than in Europe, thus preventing the assumption of global “trends” in infection. Nonetheless, canine angiostrongylosis is considered endemic in certain areas of Europe, including regions of Denmark, Germany, Hungary, Finland, France, Ireland, Italy, the Netherlands, Poland, Slovakia, Spain, Sweden, Switzerland, Turkey and the United Kingdom, in Canada (Newfoundland), in South America (Brazil and Colombia), and in Uganda in Africa. The country list is growing as the *A. vasorum* geographic distribution continues to evolve in several countries of Europe [31], and there is a growing risk of establishment in the Americas [32]. Studies carried out within dog populations in the United Kingdom, Denmark, Germany and Greece estimate the prevalence of *A. vasorum* to range from 0.3-9.8%. Estimated prevalence varies widely between different canine populations (e.g. pet, hunting or stray dogs), health status (e.g. clinically affected or healthy), and methods used (e.g. variations on coprological methods). This is in contrast to studies within fox populations in Canada, Denmark, Hungary, Italy, and Spain, which estimate prevalence of *A. vasorum* to be often higher at 5-56% [1]. These data suggest that foxes are the most important reservoir of infection, but must be interpreted with care, as figures exclude the recently recognised shifts in prevalence both within and outside of regions of endemic foci [6].

### 4.1. Pattern of spread

Information on the exact geographic range of *A. vasorum* in dogs is lacking due to apparent expansion both around known endemic foci and into previously free regions. Attention should be drawn to the fact that the lack of evidence of *A. vasorum* in a given region does not ensure its non-existence, and therefore, geographic location should not be used as the sole criterion to suspect or rule out diagnosis. Likewise, increasing awareness of the parasite among clinicians and parasitologists has driven surveillance and recognition of infection in an expanding area, some of which might have been pre-existing. However, this is unlikely to fully explain apparent range expansion, given that such a highly pathogenic disease would not lie unrecognised for so long in countries with a well-developed veterinary clinical sector. A caveat worth mentioning here is that this disease may go unnoticed unless clinicians are looking for it or test for it since making a clinical diagnosis can be difficult because of varying magnitudes of severity and a variable and sometimes vague presentation.

In Europe, *A. vasorum* is known to be spreading into previously un-infected areas in Denmark, Germany, Greece, the Netherlands, Ireland, Italy, Sweden and the United Kingdom [6,10,11,33–36]. In the British Isles, *A. vasorum* infection was first reported in a Greyhound in Ireland in 1968 [37] and 7 years later a single infection was reported in England in a Greyhound that had been imported from Ireland [38]. Following this, the infection has rapidly spread to establish regions of endemic foci in the South-West, as well as the South-East of England and Wales. Furthermore, subsequent studies have identified new pockets of *A. vasorum* infection in both dog and fox populations reaching the North of England [39], Scotland [40] and Ireland [41]. This pattern of expansion appears to be repeated at a range of scales, and in some cases the disease is appearing in different countries, for example, *A. vasorum* infection has been reported for the first time in a native dog in Slovakia in 2013, [42], the latest in a long series of new national records in Europe.

In North America, the situation is different to that seen in South America and Europe. Until recently, Newfoundland in Canada was regarded as the only *A. vasorum* endemic location in North America [9]. However, an autochthonous infection has been recently identified in the lungs of a red fox from West Virginia, USA [15]. Two cases of natural *A. vasorum* infection had been previously reported in domestic dogs in the USA; one at Michigan State University in 1985, in a Greyhound imported from Ireland [43] and the other in an abandoned dog in Long Island, based on the tentative identification of *A. vasorum* in the faeces [44].

**Figure 4:**
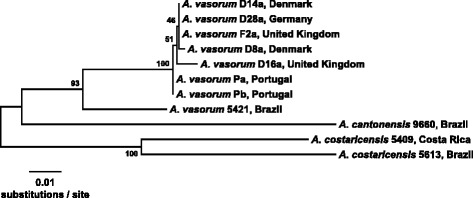
**Phylogenetic relationships among three species of** ***Angiostrongylus*** **.** Neighbor joining analysis was conducted on the concatenated sequences of COI and ITS-2 using the Kimura two-parameter model of nucleotide substitution. Bootstrap values based on 500 replications are given at the internal nodes. Taxa are labeled with the species, strain designation, and country of origin for each isolate.

It is difficult to say why *A. vasorum* has spread in the manner that it has, and why it is moving to different locations. Three of the more commonly proposed hypotheses are climate change, urbanisation of red fox populations and increased pet movements. Other equally plausible explanations include the increasing awareness of *A. vasorum* within the veterinary profession and continually improving, more readily available diagnostics. A recent report, analysing the genetic sequence in nematodes recovered from Europe and South America, questioned previous assumptions about the worldwide spread of *A. vasorum* from France. It is likely that *A. vasorum* in Europe and South America occurred as a result of evolutionary divergence along with canid radiation (Figure 4), rather than recent historical spread [13,45].

### 4.2. Risk factors and at risk-populations

Published data in the United Kingdom suggest that some purebred dogs are at higher risk than crossbreeds, and in particular Cavalier King Charles Spaniels and Staffordshire Bull Terriers [8] and beagles [9]. Reports of breed predisposition might reflect popular breeds in the locations assessed. However, data from Canada suggest a higher prevalence in hunting dog breeds [9,32], and data from The Netherlands, Germany, Denmark and Italy do not report breed preferences [10,11,34]. The most likely age for infection has been reported as around 10 months [7,8]. There are no data to suggest that age, sex or body condition influence *A. vasorum* infection in foxes [5].

### 4.3. Role of wildlife reservoirs

Besides the natural definitive host (domestic dog), *A. vasorum* has been reported from a wide range of wild canids in several geographic areas (reviewed in [1,14]). The lack of genetic separation between *A. vasorum* obtained from dogs, foxes and coyotes supports the hypothesis that transmission can occur between wild and domestic canids [13]. Indeed, infection in dogs seems to occur due to geographic over-lap with wild canid hosts [14].

The most important and best-studied wildlife reservoir for *A. vasorum* infection is the red fox (*Vulpes vulpes*). A possible explanation for increased transmission of infection between red fox and dog populations is an increasing density of foxes. It can be assumed that an increased density of foxes in an area populated with dogs is likely to increase the number of fox-dog interactions, and hence increase the opportunity for transmission of infection. Direct wild canid-dog interaction is not necessary for (and does not lead to) transmission of this parasite from one to the other because transmission occurs via ingestion of L3 (in gastropods or frogs, or possibly free in the environment). An unrelated disease outbreak could decrease the overall health of foxes, and hence increase susceptibility to *A. vasorum* infection. This could have been the case in the United Kingdom, where *A. vasorum* infection appeared simultaneously with an outbreak of sarcoptic mange [14], although this outbreak was also associated with a marked decrease in fox population density, which should have reduced *A. vasorum* infection pressure.

**Figure 5:**
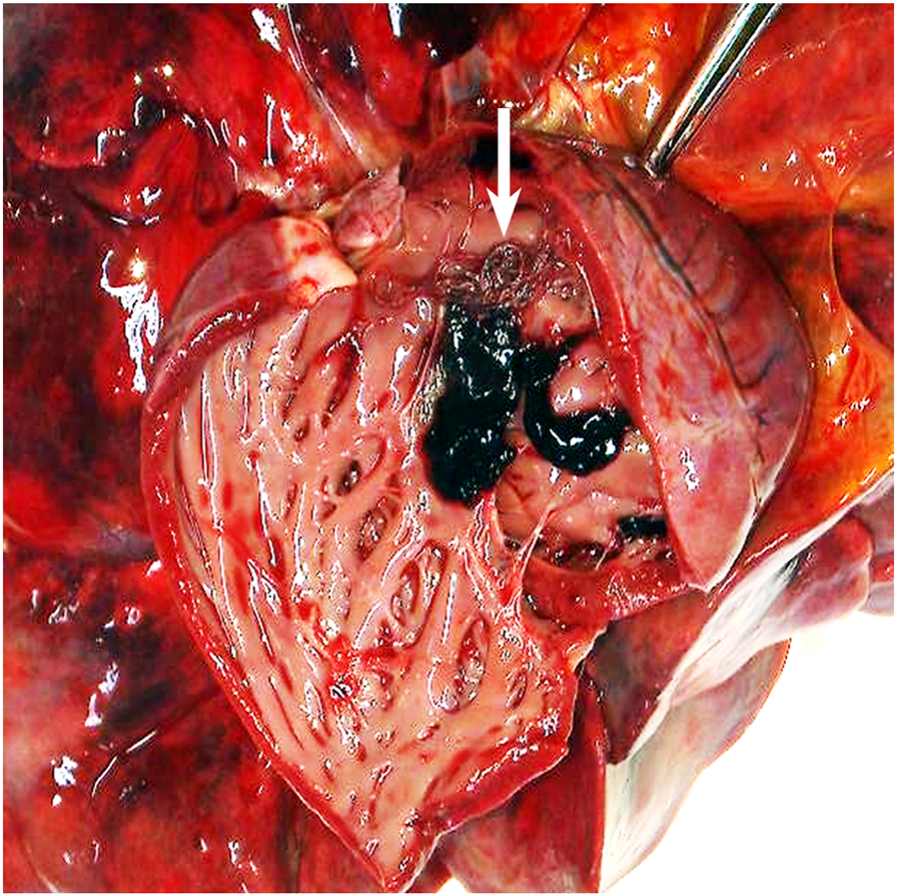
**Photograph showing** ***Angiostrongylus vasorum*** **in situ.** Adult male and female *A. vasorum* (arrow) in the heart of a red fox from the East Midlands region, England.

Studies in the United Kingdom have demonstrated a prevalence of 23.2% *A. vasorum* infection in fox populations residing in a known endemic hotspot for dog infection, compared to a countrywide prevalence of 7.3% [5]. Interestingly, we identified one case of *A. vasorum* in red fox in the Midlands of England (Figure 5), which may explain the recently identified cases of canine infection in that locality [4,46]. This finding would correspond with the situation in Canada, whereby *A. vasorum* infection was first identified in red foxes in Newfoundland in 1973, where the parasite is still endemic [47], before it emerged in domestic dogs in 1996 and then in coyotes in 2003 [25,48–50]. *A. vasorum* was recently identified in foxes in Scotland [51] where a single case of *A. vasorum* had been identified in a dog that had never travelled [40].

While *A. vasorum* is commonly found in and known to cause pathology in red foxes [5,52], only two cases have been described in wolves, first in northwestern Spain [23] and recently in Rome, Italy [24], again coinciding with increasing records of *A. vasorum* in dogs and foxes in the same geographic region, where infection with the parasite is also expanding in dogs [31,36]. Eurasian badgers, *Meles meles* (Linnaeus, 1758) have been identified as potential reservoirs for infection, however their role in transmission to dogs remains uncertain [26]. Much remains to be understood about the dynamics of infection in wild populations and factors underlying spill-over to dogs.

## 5. Disease clinical features

Infection with *A. vasorum* may be asymptomatic in some cases or may result in a wide spectrum of clinical signs, ranging from mild respiratory manifestations to severe forms [1], which are characterized by coagulative, respiratory or neurological disorders [53]. Respiratory signs are the most common clinical manifestations, with coagulation disorders being less common but more likely to be fatal. Ocular signs, including intraocular larval migration, while rare, have been reported [54].

### 5.1. Pulmonary

**Figure 6:**
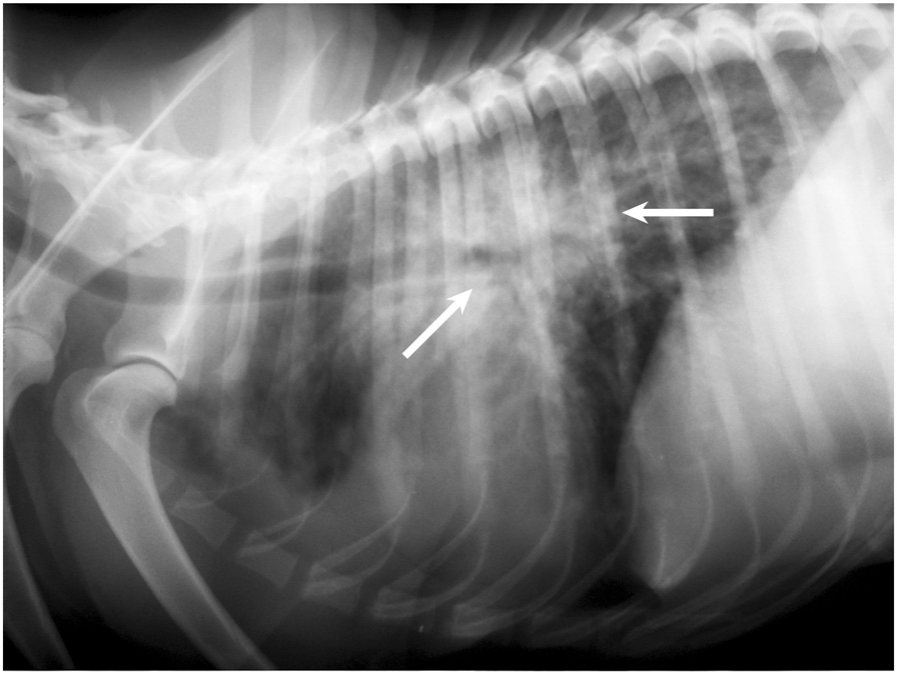
**Radiographic feature of** ***Angiostrongylus vasorum*** **.** Latero-lateral thoracic radiograph of an 18 month old English Setter, before treatment for *Angiostrongylus vasorum*. Diagnosis was confirmed by faecal analysis. Note the marked bronchial-alveolar pattern (arrows).

By far the most common clinical presentations in dogs diagnosed with *A. vasorum* are pulmonary related. The most significant of these are coughs (either productive or unproductive), and dyspnoea, with or without tachypnoea. Related clinical signs that are less commonly reported are intolerance to exercise and lethargy. It is expected that a dog positive for infection with *A. vasorum* would show some pulmonary changes upon lateral or ventrodorsal radiography (Figure 6). General findings include increased interstitial, peribronchial and alveolar patterns, pneumothorax [16,55,56], subcutaneous emphysema and an abnormally wide cranial mediastinum. The most common signs noted upon radiographic analysis are an alveolar infiltrate and bronchial thickening. Characteristic serpiginous/circular areas of radiopacities have been detected in asymptomatic dogs, which were attributed to fistulas developed during migration of larvae from the pulmonary capillaries into the alveoli [57]. There is some evidence as to the time following infection that clinicians should expect to see radiographic changes. It has been suggested that five to seven weeks after infection a bronchial-interstitial pattern can be observed, which will be most obvious at nine weeks post infection, and decrease up until approximately twenty one weeks when evidence of fibrosis will be seen [7]. Correlation between time and radiographic pattern can be followed in experimentally infected dogs [58] using computer tomography. It is evident that there are always fibrotic tissues left, showing that after *A.vasorum* infection the lesions cannot fully regress.

### 5.2. Coagulopathy

A less common but often more severe consequence of infection is a varying degree of coagulopathy [14]. Despite recent interest in this aspect of infection there is actually very little evidence based knowledge of how or why coagulopathy occurs. Coagulopathy when present can be wide ranging with some animals presenting with clinical or clinico-pathological signs more suggestive of primary (e.g. Disseminated Intravascular Coagulation (DIC) [59], immune-mediated thrombocytopenia [60,61]), secondary and tertiary coagulopathy (dysregulation of the anticoagulation pathway [59,62]) or combinations of the three. Abnormalities attributed directly to coagulopathies include haematoma, anaemia, intracranial haemorrhage, sub-conjunctival haemorrhage, melaena and increased post traumatic bleeding tendencies.

### 5.3. Cardiovascular

Cardiovascular abnormalities associated with infection include; attenuated cardiac sounds, myocarditis, heart murmurs of grade II to VI, heart failure, periarteritis, pale mucous membranes and haematoma (causing pulmonary hypertension or right heart failure secondary to obstruction) [8,60]. Pulmonary hypertension occurs in less than 5% dogs infected with *A. vasorum* in primary practice but affects up to one third of infected dogs presenting at referral practice, documented by echocardiography [1]. Echocardiology of dogs positive for *A. vasorum* has indicated dilation of the right ventricle and atrium, a bulging interatrial septum, dilated vena cava and hepatic veins, and a severe tricuspid incompetence [63,64]. Some abnormalities identified upon cardiac radiography include right ventricular enlargement, truncated pulmonary arteries and an increase in vertebral heart scale. Cardiac abnormalities are relatively rare and therefore not that commonly diagnosed, in some cases Doppler ultrasound is used as a supplementary method [7,63].

### 5.4. Nervous

Neurological clinical presentation of *A. vasorum* infection is less frequent than cardio-vascular and pulmonary, but is still of clinical importance. Most neurological signs arise from bleeding in or around the central nervous system, secondary to coagulopathies. Neurological deficits or abnormalities will reflect the area of CNS haemorrhage and prompt clinicians to consider testing for *A. vasorum* in young animals with acute onset central neurological signs before embarking on expensive and potentially risky CNS imaging whether they present with or without respiratory signs. Neurological defects associated with *A. vasorum* infection are difficult to diagnose using conventional radiographs; instead MRI and CT imaging can be used. Studies using these techniques have noted accumulation of fluid on the spinal cord, intramedullary haemorrhage and intraparenchymal haemorrhage in infected dogs [60,61,65,66]. Although nervous signs may be due to the disseminated spread of L1 no reported cases to date reflect neurological signs secondary to ectopic parasite or hypoxia as has been previously speculated [1,8,57,65,67].

## 6. Diagnosis of angiostrongylosis

### 6.1. Laboratory diagnosis

#### 6.1.1. Pathological findings

*Post mortem* reports in dogs and foxes infected with *A. vasorum* show evidence of a thickened right ventricle, with pneumonia and breakdown in alveolar structure [5,68]. Histopathological analysis typically reveals the presence of inflammatory cells, in particular neutrophils, and granulomatous foci with multinucleated giant cells, a pattern also seen in less usual definitive hosts such as the wolf [24] and coyote [25]. These observations are attributed to the inflammatory response to eggs and migrating larvae [1]. A number of studies have shown the presence of immature and adult nematodes in ectopic locations, for example in the eye [54,66,69], where they can provide a highly suggestive *ante mortem* diagnosis.

#### 6.1.2. General laboratory findings

Various haematological and biochemical abnormalities have been noted in infected dogs, including increased ß-globulin fraction, anaemia, eosinophilia, thrombocytopenia, hypercalcaemia and low serum fructosamine. However, these vary between cases, with no typical or pathognomonic profile evident [2,70,71]. While peripheral eosinophilia is not a consistent finding in dogs with angiostrongylosis, if present in an animal with appropriate clinical findings it should prompt investigation for the parasite. Coagulation parameters are also inconsistent, even in cases with clinical coagulopathies [8,59]. Thromboelastography is a useful technique, both in terms of giving additional information about the kind of coagulopathy, and also in follow-up assessment.

#### 6.1.3. Parasitological findings

The most common method for diagnosing *A. vasorum* infection is the Baermann migration-sedimentation technique, in which larvae are recovered from faeces and identified based on morphological features. Larvae have a characteristic sinusoidal terminal appendage that is quite unlike other parasitic and non-parasitic nematodes commonly found in dog faeces [72]. However, the fact that larvae cannot be found in the faeces during the pre-patent period, and the limited sensitivity of the test on a single faecal examination, are important limitations of the test [73]. This has led to modifications attempting to improve the diagnostic value of faecal larval analysis [74]. Sensitivity can be improved by pooling faeces over a three day period [1], but this and the fact that the Baermann test can take up to 24 hours to yield results, makes it onerous in clinical practice. Early examination of the Baermann’s test leads to more rapid results but at the cost of sensitivity, while direct smear examination of faeces is a simple test to perform at the pet-side but with a low sensitivity of up to 54-61% [6,75]. L1 may be detected in faecal flotation preparations, but do not float reliably in standard flotation solutions, and so are unlikely to be found in routine screening tests for parasite ova. FLOTAC, an improved flotation-based coprological method for visualising parasite eggs, oocysts and larvae in faecal samples, has been found to be more sensitive than the Baermann method for detecting *A. vasorum* L1 when used with higher specific gravity solutions [76]. Bronchoalveolar lavage (BAL), in which fluid is injected into and then recovered from the lungs and analysed for the presence of larvae, can be useful to demonstrate infection [77], but is difficult and not without risk, especially in dyspnoeic patients. It is worth mentioning, however, that evidence for the utility of BAL in the diagnosis of angiostrongylosis [77] was based on experimental findings and, hence might not accurately reflect performance in clinical cases.

### 6.2. Immuno-diagnosis

#### 6.2.1. Antigen-based assays

Detection of circulating parasite antigen was identified as a promising diagnostic approach [73], and an antigen blood test is now commercially available in the form of an in-hospital lateral flow device (AngioDetect®, Idexx Laboratories, USA). The test can be performed on plasma or serum with results obtained in 15 minutes. Antigen detection by Enzyme-linked immunosorbent assay (ELISA) has a high specificity (94%) and sensitivity (95%), much higher than faecal examination [78]. As such, this is likely to be increasingly relevant to clinical diagnosis in practice, as well as monitoring of parasite distribution and spread [79]. Baermann can still be a reliable method for diagnosis of *A. vasorum*, if performed appropriately.

Antigen is reported to be detectable around five weeks after experimental infection, hence during the pre-patent period, and to persist until after elimination of the parasite, while cross-reactions with other common canine parasites were not found [78]. Commercial antigen-detection kits for *Dirofilaria immitis*, on the other hand, were tested with sera from dogs infected with *A. vasorum*, and some cross-reactions were detected [80]. Given the emergence of *A. vasorum* in areas endemic for *D. immitis* [15], this is of some concern. It is also worth mentioning that initiation of adequate treatment has been shown to decrease the level of detected circulatory antigens. No circulating antigen was observed in dogs treated with imidacloprid/moxidectin at 4 or 32 day post infection (dpi), while in dogs treated at 88-92 dpi, circulatory antigens decreased within 13-34 days [78]. Antigen detection could therefore be useful as an indicator of response to treatment.

#### 6.2.2. Antibody-based assays

Detection of specific antibodies against *A. vasorum* also hold potential for clinical diagnosis [81]. However, a study using Western Blot and ELISA serology of 14 dogs experimentally infected with *A. vasorum,* successfully identified only five animals over a period of 0-56 dpi [82]. Other attempts to identify antibodies, based on crudely extracted parasite antigens, suffered from lack of specificity [83]. Further work has been carried out to identify specific proteins produced by both L1 and adult nematodes, that are recognised by antibodies, in order to improve the sensitivity of such tests [84,85]. A novel antibody-detection ELISA, based on monoclonal antibodies to *A. vasorum*, succeeded in achieving high sensitivity and specificity [80], and has been applied alongside antigen-detection in serological surveys [46,79]. In theory, persistence of antibodies in dogs historically infected could lead to false positives when using antibody detection as a clinical diagnostic tool, especially in areas with high background levels of exposure. Hence, antibody testing alone is of limited value except as a means of screening for exposure in presumably non-endemic regions. The options for combining antigen and antibody detection ELISA in clinical and epidemiological settings are discussed in Schnyder et al. [46]. There may also be some potential for combining an antibody detection ELISA with the Baermann technique to increase the latter’s sensitivity but retaining the early detection benefits of the former.

### 6.3. Molecular techniques

Polymerase chain reaction (PCR) has been used to successfully recognise unique nucleic acid sequences of *A. vasorum* [40]. Real-time PCR was developed for *A. vasorum* in definitive and intermediate hosts, and shown to be capable of detecting DNA circulating in canine blood [81,85]. Although highly specific and requiring a blood sample rather than faeces, real-time blood PCR offered no great advantage over the Baermann test in terms of sensitivity in naturally infected dogs [85]. PCR has also been used to detect *A. vasorum* in the faeces of dogs [83] and foxes [86]. However, PCR inhibitors in faeces can limit sensitivity and there is no obvious advantage over the Baermann test unless faeces are to be stored for long periods prior to examination, such that L1 die and are unable to migrate in the Baermann apparatus. PCR can also be useful in cases where faecal Baermann is not suggestive of a diagnosis of L1 of *A. vasorum* because they are morphologically altered or less motile than usual. However, the effect of treatment (e.g. fenbendazole) on the size or motility of the larvae prior to faecal screening remains unknown or poorly understood. PCR may be also useful in instances where a clinician is faced with a case that clinically resembles *A. vasorum* (i.e. respiratory signs with either clinical or clinico-pathological evidence of bleeding diathesis) but faecal analysis is not supportive.

### 6.4. Problems in diagnosing angiostrongylosis

Clinicians are faced with many diagnostic challenges when it comes to definitively identifying *A. vasorum* infection in a patient. In this regard several points are worth mentioning. Firstly, due to the recent erratic and somewhat unexplained spread of infection, from the classified ‘hotspots’ to previously uninfected areas, it is no longer possible to reliably determine whether an animal is at a high or low risk of infection according to its location. Secondly, it is possible that a clinician may overlook clinical signs suggestive of *A. vasorum* infection, and treat for a condition with a similar presentation such as kennel cough, because of a belief that *A. vasorum* is not present in the area. Thirdly, it is widely accepted that tests such as Baermann faecal examination do not always recognise the presence of *A. vasorum* in an animal, especially in pre-patent infection or as a result of low larval loads, intermittent larval shedding, poor sample quality or operator error. Finally, the low performance of faecal tests in terms of sensitivity and immediacy has led many clinicians to treat presumptively for angiostrongylosis in suspicious cases, rather than pursue a definitive parasitological diagnosis. This has the disadvantage of removing an important source of evidence to the clinician on local level of risk, as well as monitoring of treatment success. Blind treatment, without attempts to make a diagnosis, in cases of high suspicion should be discouraged for this reason and because the required duration of treatment is dependent on the animal not continuing to shed L1. Wider availability of pet-side antigen-detection tests is likely to provide important clinical and epidemiological information in the future, on which decisions on treatment and prevention regimes can be based.

## 7. Treatment

*A. vasorum* is susceptible to a variety of anthelmintics. Historically levamisole and ivermectin were routinely used but this has not been the case in the past decade. This is in part due the risk of anaphylaxis with levamisole use [87], the risk of toxicosis with ivermectin use in any breed with an MDR 1 mutation (e.g., collie breeds), and, most importantly, safer and licensed products becoming available. Fenbendazole remains a popular treatment of choice and is efficacious at 25-50 mg/kg for 5-21 days [1,12,88], but in many territories is not licensed as a treatment for *A. vasorum*. The reasoning behind fenbendazole use is that it produces a “slow kill” and therefore reduces the risk of anaphylaxis but there remains no peer reviewed data to support this theory. There are currently only two widely licensed parasite treatments that include *A. vasorum* among their label claims. One is a moxidectin/imidacloprid spot-on solution (Advocate®, Bayer Animal Health) and the other a milbemycin oxime tablet, in combination with praziquantel (Milbemax®, Novartis Animal Health). Moxidectin has been shown to be effective in removing adult *A. vasorum* and immature stages (L4 and L5) and milbemycin in reducing levels of adult and immature adult (L5) infection [12,89]. Moxidectin requires a single monthly spot-on application to eliminate infection. Milbemycin requires weekly oral administration for 4 weeks to treat clinical *A. vasorum* infection. Both are highly efficacious treatments. The study comparing the efficacy of moxidectin/imidacloprid to fenbendazole [12] as a treatment option actively excluded severe cases of angiostrongylosis and therefore little is known about this drug’s performance in severe cases. Milbemycin was also recently shown to have 98.8% preventative efficacy against development of adult *A. vasorum* infection in a combination product with spinosad (Trifexis®, Elanco Animal Health) but at the time of writing this combination is not yet licensed for *A. vasorum* treatment and prophylaxis [90]. Licensed claims often differ between territories and change with time, and readers are advised to check on the current situation in their locality before making recommendations on treatment. Supportive treatment is often indicated, depending on the clinical presentation. Anti-inflammatory doses of steroids have been used to moderate potential anaphylaxis and/or treat significant respiratory signs [54]. Oxygen administration is also indicated in the respiratory compromised patient. Patients with coagulopathies have received lifesaving treatment with coagulation factors delivered in fresh frozen plasma or whole blood, although cases with coagulopathies may recover also with treatment of the parasite alone [63]. Where congestive heart failure is present as a result of infection then treatment with diuretics, ACE inhibitors and phosphodiesterase inhibitors (e.g., pimobendan or sildenafil) for pulmonary hypertension, are indicated.

## 8. Control and prevention

Eradication of *A. vasorum* is impractical in any given area since significant reservoirs of infection will almost certainly be present in the wild fox and intermediate host populations. Measures should be considered to avoid dogs consuming L3 (third stage larvae) either directly or in gastropod molluscs. These include feeding dogs indoors, cleaning outdoor bowls and toys frequently (or making them less accessible to slugs/snails) to reduce the risk of contamination with L3.

While molluscicides can be employed to reduce slug and snail abundance, many of these are not pet safe and to prevent access of pet dogs to molluscs completely is unrealistic. Infected molluscs killed by pesticides in gardens might be more available to dogs and actually increase risk of infection. Correct disposal of dog faeces, while being desirable for control of parasites such as *Toxocara canis*, is unlikely to make a significant impact on environmental contamination with L1 as current data suggest that domestic dogs are a much smaller reservoir of infection when compared to foxes; nevertheless, treatment of dogs and faecal removal might enhance local protection, for example if environmental contamination is predominantly from dogs in one locale, or in public areas highly frequented by dogs. An interesting recent study has experimentally explored the use of nematophagous fungi in the environmental control of *A. vasorum*, by demonstrating an efficacious destruction of L1 [91], but currently anthelmintic prophylactic treatment remains the mainstay of disease prevention. Education is essential to raise public awareness as canine angiostrongylosis continues to be seen with increasing frequency in regions where it was not traditionally thought to be endemic and it should be considered an emerging disease of which veterinarians should be aware. Moxidectin and milbemycin oxime can be used effectively for *A. vasorum* prophylaxis if used monthly [89,90]. So far, no report has been published on resistance of *A. vasorum* to chemotherapeutic compounds and large intermediate host and wildlife reservoirs are expected to buffer against resistance. However, long term excessive treatment may potentially favour the development of anthelmintic resistance and as a result the need for monthly prophylaxis should be based on risk factors such as whether the patient is known to come into frequent contact with slugs or snails or has had previous exposure to the parasite. Prophylactic treatment should be initiated in all dogs having suffered from *A. vasorum* infection as it would imply that the area is endemic and the dog is coming into contact with the intermediate host. Alternatively, dogs may be regularly checked for the presence of *A. vasorum*, something that is easier with the availability of the new diagnostic tests described above.

## 9. Conclusions

On any ranked list of cardiopulmonary nematode parasites of veterinary importance, *A. vasorum* would have to lie towards the top. In recent years the threat of *A. vasorum* to companion animal populations has become increasingly apparent. This parasite has considerable impact on dogs’ health and has shown both regional endemicity and geographic expansion. Going from its original discovery in France in the 1800s [3], to the recent cutting-edge elucidation of the *A. vasorum* proteome [92], mitochondrial genome [93] and transcriptome [94] considerable insights into understanding of the contribution of virulence determinants to the pathophysiological aspects of canine angiostrongylosis have been gained. However, important gaps remain in the available literature surrounding the epidemiology of infection. It would be useful to establish why infection is so prevalent in hotspots around the world and the factors that cause infection to spread. The role of red foxes and other wild canids in the transmission cycles of *A. vasorum* is not adequately understood. Also, the prevalence and geographic distribution of *A. vasorum* among wild canids, as well as how *A. vasorum* was introduced to new localities (e.g. the USA) warrant further epidemiological investigation. Attention should be drawn to the fact that the lack of evidence of *A. vasorum* in a given region does not guarantee its nonexistence. Thus, location should not be used as the sole criterion to suspect or rule out diagnosis. While intense investigations in multiple directions have been conducted to elucidate the mechanisms conducive to the development of coagulopathies, no consensus has yet emerged. Knowledge of this may help to reduce the number of fatal cases observed. Better control of *A. vasorum* will have implications for the health of domestic dogs. Development of a safe and efficacious canine vaccine for the prophylaxis of angiostrongylosis disease could, if feasible, be a significant tool in future prevention programmes.
